# Effects of undergraduate medical students’ individual attributes on perceptions of encounters with positive and negative role models

**DOI:** 10.1186/s12909-016-0686-1

**Published:** 2016-06-23

**Authors:** Masami Tagawa

**Affiliations:** Center for Innovation in Medical and Dental Education, Graduate School of Medical and Dental Sciences, Kagoshima University, 8-35-1 Sakuragaoka, Kagoshima, 890-8544 Japan

**Keywords:** Role model, Gender, Professional development, Formal curriculum

## Abstract

**Background:**

The use of role models (RMs) is a successful educational strategy. In formal training and other settings during undergraduate education, students have the opportunity to recognize numerous traits and behaviors of their RMs, such as teaching skills, professionalism in the clinical setting, and personal qualities. Encountering both positive and negative RMs allows medical students to learn a variety of professional norms and values. This learning process is likely influenced by a student’s developmental status, which itself is related to that student’s personal attributes and experiences. The purpose of this study was to examine graduating medical students’ perceptions of their RM encounters and their learning processes, and how these perceptions and processes are affected by their own personal attributes.

**Methods:**

Sixth-year medical students were asked to complete questionnaires in 2013 and 2014 regarding encounters with positive or negative RMs, in terms of patient relationships, clinical expertise, teaching ability, and other factors, during clinical training and other situations. Associations between gender, age, admission status, and recognition of self-achievement and joy of learning in relation to RM encounters were then analyzed.

**Results:**

Among 115 students (75 males, 40 females) who completed the questionnaires, 113 (98.3 %) and 85 (73.9 %) reported encountering positive and negative RMs, respectively. The majority of students reported encountering both positive and negative RMs in terms of relationships with patients, humanity, and teaching ability, and fewer negative RMs in terms of clinical expertise and contributions to the community. Older students, males, and those who had passed an entrance examination for bachelors reported encountering more negative RMs in terms of relationships with patients, humanity, and teaching ability than younger students, females, and general admission students. These results suggested an association between positive and negative RM encounters and recognition of self-achievement and joy of learning in formal clinical training.

**Conclusions:**

Most medical students encountered both positive and negative RMs during undergraduate medical education. These findings suggest that encounters with not only positive, but also negative RMs might facilitate student learning. Therefore, personal development appears to affect student perception of RMs.

**Electronic supplementary material:**

The online version of this article (doi:10.1186/s12909-016-0686-1) contains supplementary material, which is available to authorized users.

## Background

Role modeling is an essential and powerful educational strategy that allows medical students to obtain the attributes and values of, and develop identities as, medical doctors [1, 2]. Medical students learn from role models (RMs) by example; however, not all RMs exhibit positive professional behavior. Previous studies have investigated the behavior of positive RMs through qualitative or quantitative analysis of students [3, 4], residents [5], or clinical faculty and staff [6, 7]. Passi et al. reviewed 39 studies and categorized the attributes of positive RMs into the following three domains: clinical attributes (an excellent level of clinical knowledge and skills, patient-centered approach, humanistic behavior); teaching skills (establishing rapport with learners, creating a positive and supportive educational environment, developing specific teaching methods, being committed to the growth of the learners, teaching responsibility, providing students with ample patient interaction); and personal qualities (effective interpersonal skills, a positive outlook, integrity, good leadership skills, a commitment to excellence, dedication, honesty, politeness, enthusiasm, inspiring students) [8]. In addition, Wright et al. described an excellent RM as a physician who enjoys teaching in-house staff, commits substantial effort to teaching, and emphasizes doctor-patient relationships and psychosocial aspects of patient care in their teaching [6]. Professionalism is emphasized in medical education; therefore, other studies have investigated instances in which a lack of professionalism or ethical dilemmas were encountered by medical students [9–12] and residents [13]. Responsibility for community has also been identified as another aspect of professionalism [14].

Students observe RMs both consciously and unconsciously, and as a result, typically lose some degree of their ethical behavior or humanism [9, 15]. The development of values and the ability to learn is dependent on formal training and informal and hidden curriculum in clinical courses [16–18]. Shuval and Adler analyzed the modeling processes of medical students based on socialization theory and identified the following three patterns of value formation: *active identification*, in which students’ attitudes begin to resemble those of their RMs over time; *active rejection*, in which students’ attitudes become more dissimilar to those of their RMs over time; and *inactive orientation*, a pattern of relative passivity compared to the first two, in which students display no change in their attitude [19]. To understand the influence of RMs on value formation in medical students, individual student experiences with RMs during undergraduate medical education need to be investigated, as well as whether students witness their RMs conducting ideal or unprofessional behaviors, and which student factors, aspects of RM behaviors, and learning contexts may have affected student learning and perceptions of RMs.

The purpose of this study was to examine graduating medical students’ perceptions of their RM encounters and how their personal attributes affected their perceptions. Student encounters with their RMs are also examined in relation to their recognition of self-achievement.

## Methods

This cross-sectional study was approved by and conducted according to the guidelines and requirements of the ethics committee of the Graduate School of Medical and Dental Sciences, Kagoshima University. Among all sixth-year medical students at Kagoshima University during 2013 and 2014, written informed consent was obtained from a total of 157 who agreed to participate.

### Development of the questionnaire

A self-administered questionnaire regarding medical students’ RM encounters and recognition of self-achievement and joy of learning was developed and subsequently integrated into a questionnaire for undergraduate education.

The first part of questionnaire asked students to rate their frequency of recognition of self-achievement and joy of learning in formal clinical training courses on 6-point Likert scale as follows: 1, none; 2, 1–2 times; 3, 3–4 times; 4, 5–6 times; 5, 7–9 times; and 6, 10 times or more.

The second part of questionnaire was composed of items on whether they themselves had observed good RM behavior (positive RMs) or bad RM behavior that made them think “I will never behave like that” (negative RMs) (Additional file 1). To clarify various aspects of behaviors, actions, and attributes and make student responses more concrete, the following six categories of behaviors and attributes were selected based on previous research on RMs [6, 8–14]: a) relationship with patients; b) clinical expertise; c) humanity and personal attributes; d) lifestyle; e) teaching students and health care professionals; and f) contributions to the community. Students provided “yes/no” responses based on whether they had encountered RMs in each category during formal clinical training courses or other situations.

The questionnaires were distributed in envelopes to sixth-year students in September 2013 and July 2014 who had completed all undergraduate medical courses and had previously consented to participate in this study. Students were asked to return completed questionnaires to a drop box in the student lounge. The chi-square test, t test, nonparametric Kruskal-Wallis test [20], and logistic regression were used to analyze student responses (SPSS version 23; IBM, New York, NY).

## Results

### Demographic characteristics of the participants

A total of 115 sixth-year Japanese medical students (75 males, 40 females; 2013, 44 students; 2014, 71 students) responded to both parts of the questionnaire. The response rate was 73.2 % (males, 70.8 %, females, 82.0 %) among the 157 students who consented to participate in this study, and 53.7 % (51.0 % male, 59.7 % female) among the total of 214 students who were targeted. The mean age of the respondents was 26.4 years (age range, 23–51 years; median, 25 years) (Table 1). Thirteen participants (7 males, 6 females) who had passed an entrance examination for bachelors were admitted to the middle of the second year of the medical program. The mean age of these students (33.9 years) was 8.4 years higher than that of students who had entered into medical school through general admission.

**Table 1:** Demographic characteristics of the participants

		Age (years)				
	*n*	Mean	SD	Min	Max	Median
Total	115	26.4	4.33	23	51	25
Male	75	26.7	4.46	23	51	25
Female	40	26.0	4.09	23	39	25
General^a^	102	25.5	3.42	23	51	25
Male	68	26.0	4.05	23	51	25
Female	34	24.5	0.96	23	27	24
Bachelor^b^	13	33.9	3.51	29	39	33
Male	7	33.1	2.85	31	38	31
Female	6	34.7	4.27	29	39	35

^a^Admitted to the first year of the medical program as high school graduates

^b^Graduated from other university and admitted to the 2nd year of the medical program

### RM encounters in formal clinical training courses and other situations

**Fig. 1:**
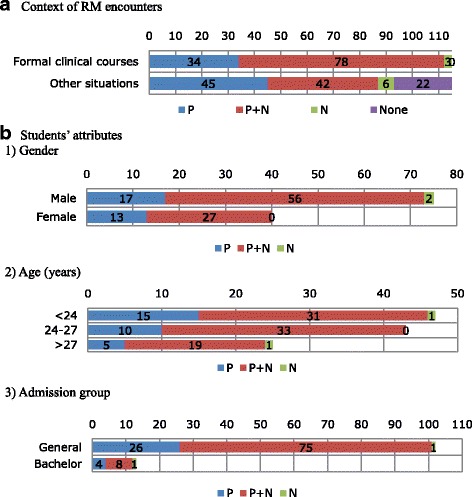
Number of students who encountered role models (RMs) during undergraduate medical education, based on the contexts (**a**) and students' attibutes (**b**). P: students who encountered only positive RMs; P + N: students who encountered both positive and negative RMs; N: students who encountered only negative RMs; None: students who did not encounter any RMs

All 115 students (100 %) indicated that they had encountered RMs in formal clinical training courses, and 93 (80.9 %) had encountered RMs in other situations (Fig. 1). A total of 113 (98.3 %) and 85 (73.9 %) students reported encountering positive and negative RMs, respectively, with 112 (97.4 %) and 81 (70.4 %) encountering positive and negative RMs in formal clinical training courses.

Regarding the experiences of each student with RMs, 83 students (72.2 %; 56 males [74.4 %], 27 females [67.5 %]) encountered both positive and negative RMs, 30 (26.1 %; 17 males [22.7 %], 13 females [32.5 %]) encountered only positive RMs, and two male students (1.7 %) encountered only negative RMs during undergraduate medical education (Fig. 1b). Students’ RM encounters by age and admission group are shown in Fig. 1. More male and older students encountered negative RMs, but this difference was not significant. Overall, no significant differences were found in positive and negative RM encounters between gender, age, or admission groups.

### Aspects of RM behavior and attributes

RM encounters were analyzed according to six RM categories. As shown in Table 2, the number of students who encountered positive RMs in formal clinical training courses ranged from 84 (73.0 %, “lifestyle”) to 104 (90.4 %, “teaching”), and the number of students who encountered negative RMs ranged from 22 (19.1 %, “community contribution”) to 73 (63.5 %, “relationship with patients”). Students reported encountering both positive and negative RMs in terms of “relationship with patients”, “humanity”, and “teaching”, and fewer negative RMs in terms of “lifestyle”. In other informal situations, the numbers of students who encountered positive RMs ranged from 61 (53.0 %, “clinical expertise”, “teaching”) to 69 (60.0 %, “relationship with patients”), and the numbers of students who encountered negative RMs ranged from 14 (12.2 %, “contributions to the community”) to 38 (33.0 %, “relationship with patients”).

**Table 2:** Numbers and attributes of students who encountered role models (RMs) by category, context, and type

RMs			Students who encountered RMs															
Category of behaviors & attributes	Context of encounter	Type Positive/Negative	Total	Gender				Age						Admission				
				Male		Female		<24		24–27		>27		General		Bachelor		
				*n*	%	*n*	%	*n*	%	*n*	%	*n*	%	*n*	%	*n*	%	
			115	75	100 %	40	100 %	47	100 %	43	100 %	25	100 %	102	100 %	13	100 %	
a. Relationship with patients	Formal	Positive	102	66	88.0 %	36	90.0 %	44	93.6 %	38	88.4 %	20	80.0 %	93	91.2 %	9	69.2 %	
		Negative	73	50	66.7 %	23	57.5 %	26	55.3 %	29	67.4 %	18	72.0 %	65	63.7 %	8	61.5 %	
	Others	Positive	69	45	60.0 %	24	60.0 %	26	55.3 %	25	58.1 %	18	72.0 %	62	60.8 %	7	53.8 %	
		Negative	38	*32*	*42.7 %*	*6*	*15.0 %*	12	25.5 %	14	32.6 %	12	48.0 %	32	31.4 %	6	46.2 %	a
b. Clinical expertise	Formal	Positive	103	68	90.7 %	35	87.5 %	41	87.2 %	40	93.0 %	22	88.0 %	92	90.2 %	11	84.6 %	
		Negative	27	18	24.0 %	9	22.5 %	10	21.3 %	10	23.3 %	7	28.0 %	25	24.5 %	2	15.4 %	
	Others	Positive	61	44	58.7 %	17	42.5 %	25	53.2 %	22	51.2 %	14	56.0 %	56	54.9 %	5	38.5 %	
		Negative	19	16	21.3 %	3	7.5 %	*4*	*8.5 %*	*7*	*16.3 %*	*8*	*32.0 %*	17	16.7 %	2	15.4 %	b
c. Humanity, personal attributes	Formal	Positive	100	64	85.3 %	36	90.0 %	42	89.4 %	39	90.7 %	19	76.0 %	92	90.2 %	8	61.5 %	
		Negative	65	45	60.0 %	20	50.0 %	23	48.9 %	23	53.5 %	19	76.0 %	57	55.9 %	8	61.5 %	
	Others	Positive	66	43	57.3 %	23	57.5 %	25	53.2 %	27	62.8 %	14	56.0 %	60	58.8 %	6	46.2 %	
		Negative	30	23	30.7 %	7	17.5 %	8	17.0 %	11	25.6 %	11	44.0 %	24	23.5 %	6	46.2 %	
d. Lifestyle	Formal	Positive	84	58	77.3 %	26	65.0 %	36	76.6 %	31	72.1 %	17	68.0 %	75	73.5 %	9	69.2 %	
		Negative	36	26	34.7 %	10	25.0 %	12	25.5 %	15	34.9 %	9	36.0 %	32	31.4 %	4	30.8 %	
	Others	Positive	64	43	57.3 %	21	52.5 %	22	46.8 %	28	65.1 %	14	56.0 %	58	56.9 %	6	46.2 %	
		Negative	19	16	21.3 %	3	7.5 %	5	10.6 %	8	18.6 %	6	24.0 %	17	16.7 %	2	15.4 %	
e. Teaching students and health care professionals	Formal	Positive	104	66	88.0 %	38	95.0 %	43	91.5 %	42	97.7 %	19	76.0 %	95	93.1 %	9	69.2 %	
		Negative	65	45	60.0 %	20	50.0 %	22	46.8 %	25	58.1 %	18	72.0 %	58	56.9 %	7	53.8 %	
	Others	Positive	61	42	56.0 %	19	47.5 %	24	51.1 %	22	51.2 %	15	60.0 %	55	53.9 %	6	46.2 %	
		Negative	22	18	24.0 %	4	10.0 %	*2*	*6.4 %*	*7*	*16.3 %*	*12*	*48.0 %*	16	15.7 %	6	46.2 %	c
f. Contributions to the community	Formal	Positive	102	65	86.7 %	37	92.5 %	43	91.5 %	40	93.0 %	19	76.0 %	91	89.2 %	11	84.6 %	
		Negative	22	16	21.3 %	6	15.0 %	6	12.8 %	10	23.3 %	6	24.0 %	20	19.6 %	2	15.4 %	
	Others	Positive	64	42	56.0 %	22	55.0 %	24	51.1 %	25	58.1 %	15	60.0 %	57	55.9 %	7	53.8 %	
		Negative	14	13	17.3 %	1	2.5 %	*2*	*4.3 %*	*5*	*11.6 %*	*7*	*28.0 %*	12	11.8 %	2	15.4 %	d

Formal: RM encounters in formal clinical courses

Others: RM encounters in informal situations

a-d: Logistic regression was applied to predict RM encounters by category, context, and type (positive or negative) (independent variable) with gender, age, and admission group as independent variables. Significant variables (italic) in the equations were gender (a) and age (b,c,d)

^a^R2(Nagelkerke)0.144, Predicted percentage correct 67.8, Significant variable in the equation (gender, sig. <0.01, Exp(B) 4.266)

^b^R2(Nagelkerke)0.153, Predicted percentage correct 83.5, Significant variable in the equation (age, sig. <0.05, Exp(B) 2.814)

^c^R2(Nagelkerke)0.248, Predicted percentage correct 80.0, Significant variable in the equation (age, sig. <0.01, Exp(B) 3.310)

^d^R2(Nagelkerke)0.144, Predicted percentage correct 87.8, Significant variable in the equation (age, sig. <0.05, Exp(B) 3.287)

### Influence of student characteristics on perceptions of RMs

Specific RM encounters (category, context, positive or negative) were also analyzed in terms of gender, age, and admission status (Table 2), as well as individual student experiences (positive RMs only, positive and negative RMs, negative RMs only, and no RM encounters) (Table 3).

**Table 3:** Student RM encounters in each category in formal clinical training courses

Encountered RMs	Students’ experience		Student attributes			
Category of behaviors & attributes			Age (years)		Gender	Admission
	Pos/Neg	*n*	Mean	SD	Male %	Bachelor %
a. Relationship with patients	P	40	26.2	4.01	62.5 %	12.5 %
	P + N	62	26.4	4.66	66.1 %	6.5 %
	N	11	28.0	3.80	81.8 %	36.4 %
	None	2	24.5	0.71	0 %	0 %
b. Clinical expertise	P	81	26.3	3.85	67.9 %	12.3 %
	P + N	22	27.2	6.20	59.1 %	4.5 %
	N	5	26.6	3.64	100 %	20.0 %
	None	7	25.4	3.41	28.6 %	14.3 %
c. Humanity, personal attributes	P	46	25.8	3.85	60.9 %	10.9 %
	P + N	54	26.6	4.62	66.7 %	5.6 %
	N	11	29.1	4.76	81.8 %	45.5 %
	None	4	24.5	0.58	50.0 %	0 %
d. Lifestyle	P	59	26.4	4.16	69.5 %	15.3 %
	P + N	25	26.7	5.88	68.0 %	0 %
	N	11	28.2	4.05	81.8 %	36.4 %
	None	20	25.2	1.99	40.0 %	0 %
e. Teaching students and health care professionals	P	49	25.9	3.98	59.2 %	12.2 %
	P + N	55	26.5	4.54	67.3 %	5.5 %
	N	10	28.8	4.57	80.0 %	40.0 %
	None	1	24.0		100 %	0 %
f. Contributions to the community	P	84	26.0	3.68	63.1 %	11.9 %
	P + N	18	27.6	6.72	66.7 %	5.6 %
	N	4	27.5	3.51	100 %	25.0 %
	None	9	27.7	4.30	66.7 %	11.1 %

*RM* role model, *SD* standard deviation

P: students who encountered only positive RMs; P + N: students who encountered both positive and negative RMs; N: students who encountered only negative RMs; None: students who did not encounter any RMs

Male or older students reported encountering negative RMs in all categories in both contexts more frequently than female or younger students (Table 2). Bachelor admission students reported encountering fewer positive RMs in all categories in both contexts, and more negative RMs in terms of “humanity” and “teaching” in other informal situations compared with general admission students.

To examine the effects of multiple factors on RM encounters, logistic regression was applied using gender, age, and admission group as independent variables. Gender was a significant factor in predicting negative RMs in terms of “relationship with patients” (percentage of correct predictions, 67.8 %; *p* < 0.01), and age was a significant factor in predicting negative RMs in terms of “clinical expertise” (83.5 %; *p* < 0.05), “teaching” (80.0 %; *p* < 0.01), and “contributions to the community” (87.8 %; *p* < 0.05) in informal situations.

Some tendencies were found regarding individual student experiences of RM encounters (Table 3). For example, students who encountered negative RMs but no positive RMs tended to be older, male, and bachelor admission students than students who encountered positive RMs; however, no significant differences in age were detected by the t test.

### Student’s recognition of achievement and RM encounters in formal clinical training courses

**Fig. 2:**
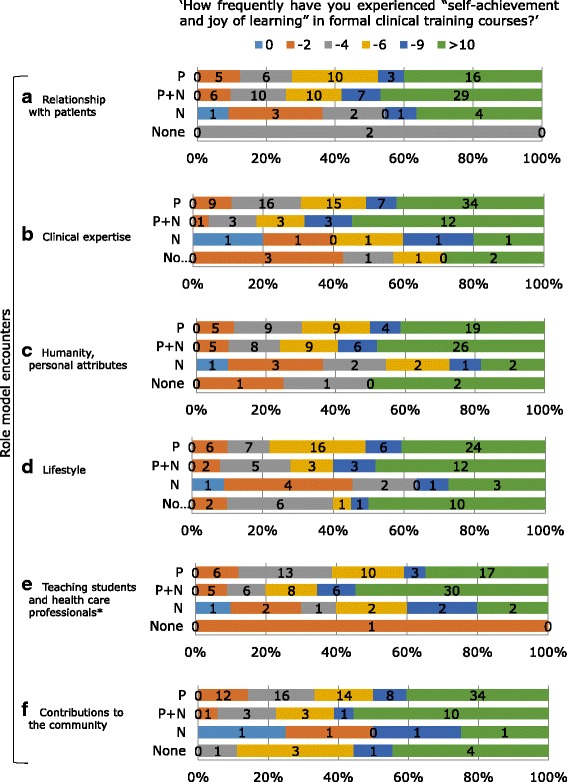
Student recognition of self-achievement and joy of learning in specific RM encounters (**a**-**f**). Students were divided into the following four RM encounter groups: P, students who encountered only positive RMs; P + N, students who encountered both positive and negative RMs; N, students who encountered only negative RMs; and None, students who did not encounter any RMs. Students rated their frequency of recognition of “Self-achievement and joy of learning” on 6-point Likert scale as follows: 1, none; 2, 1–2 times; 3, 3–4 times; 4, 5–6 times; 5, 7–9 times; and 6, 10 times or more. The number of students in each scale is indicated by the bars. *The level of significance was set at *p* < 0.05 for the Kruskal-Wallis test regarding student distribution on the scale for role model encounter groups (P, P + N, N, None)

The association between students’ “recognition of self-achievement and joy of learning” and their experiences regarding RM encounters in each category is shown in Fig. 2.

The only significantly different frequency distribution for self-achievement and joy of learning among RM encounter groups was for “teaching”; this was likely the result of the small sample size in this study. However, among all RM categories, students who encountered positive RMs (positive RMs only, positive and negative RMs) recognized self-achievement and joy of learning more frequently than those who did not encounter any positive RMs (negative RM only, no RM encounters) (difference of means for “relationship with patients” and “lifestyle”, *p* < 0.05). In addition, students who encountered both positive and negative RMs recognized self-achievement and joy of learning more frequently than those who encountered positive RMs only (difference in means for “teaching”, *p* < 0.05).

## Discussion

This study attempted to examine how graduating medical students view their previous encounters with RMs based on their values and norms and a contextual understanding, as well as which behaviors and attributes of their instructors or others encouraged students to perceive them as RMs in formal clinical training or other situations.

Although the vast majority of students reported encountering RMs who exhibited desirable actions, behaviors, or personal attributes, 74 % also reported encountering negative RMs during their undergraduate medical education. Wright reported that 88 % of the medical residents at McGill University in Canada had a personal RM [5], and each student nominated an average of 3.7 physicians as their personal RM [3]. Previous reports from the 1990s revealed that 90 % of medical students [10] and 92.5 % of residents [13] in the US either observed or experienced unethical or unprofessional conduct among their colleagues and superiors during training. In a study by Reddy et al., over 80 % of students reported receiving insufficient feedback from faculty or residents, which was recognized as unprofessional behavior [12]. In this study, a smaller percentage of students reported encountering negative RMs compared with findings from studies conducted in North American countries; this may be because of cultural differences and social norms, as Japanese medical students tend to accept professional values without being critical, or because for some reason, fewer unprofessional RMs are employed by Kagoshima University. Reddy et al. reported that medical students’ perceptions of unprofessional behavior changed during the clinical training period, as they tended to be more accepting of unprofessional behavior after they had completed clinical training [12]. Another explanation could be that general admission students who entered medical school as high school graduates were younger and found it easier to accept new values than medical students in North America, who are more similar to the bachelor students in this study. However, the majority of students in this study still encountered negative RMs, and this suggests that young trainees commonly encounter both positive and negative RMs in the clinical training environment; these encounters therefore appear to be part of the natural process of professional development.

Hafferty and Franks reported that physicians start to develop an identity of self-starting with the student selection process, and that the overall process of medical education is important for their moral training [16]. White et al. suggested that students’ pre-existing values might intensify, transform, or be compromised based on encounters with positive or negative RMs [21]. Kegan’s “self-authorship” schema explains that individuals with a developed sense of self have a “self-authorship” process characterized by being able to self-initiate, self-correct, and self-assess without necessarily having to depend on others to frame problems or articulate necessary adjustments [22].

Most students who encounter negative RMs tend to experience value conflicts [23]. Resolving value conflicts is a challenging process of value negotiations and adjustment, and this varies among students based on their developmental stage.

In this study, students who encountered both positive and negative RMs appeared to gain a greater sense of self-achievement and receive more enjoyment from learning than students who did not. Among the categories and types of RM behaviors, encountering positive RMs in terms of “relationship with patients” and “lifestyle” and both positive and negative RMs in terms of “teaching” significantly increased students’ recognition of self-achievement. Therefore, encountering multiple or different types of RMs might facilitate student learning and help them resolve value conflicts. Encountering negative RMs might have favorable effects if students’ norms and values are intensified by encountering positive RMs.

Negative RMs were less frequently associated with clinical expertise and contributions to the community. This finding is consistent with those in previous studies on unprofessional behavior, poor doctor-patient relationships, personal attributes, and teaching ability [9, 11–13]. White et al. reported that what students learned about patient-centered care during the preclinical years differed from what they actually experienced in their clinical clerkships, and suggested that their values might have been reinforced, compromised, or transformed as a result of this conflict [21]. In Japan, preclinical education regarding the patient-centered approach and teamwork may create discrepancies in values between medical students and doctors in relation to communication and interpersonal skills, which have been emphasized since the 1990s.

Students in this study reported encountering more negative RMs in relation to patient relationships and humanity compared with clinical competency, suggesting that “negative” perceptions may develop as an outcome of formal preclinical curriculum that aims to foster patient-centeredness and interpersonal skills, in addition to their values, which may be similar to those of a layperson. Furthermore, RM behaviors and attributes in relation to patient relationships, teaching, and lifestyle were influential categories for student learning, suggesting the importance of these RM behaviors and attributes for students.

A primary mission of Kagoshima University is to foster health professionals who are responsible for community medicine, including remote islands in Kagoshima Prefecture. The formal curriculum for sixth-year medical students includes a clerkship in either the remote islands or a rural area. The majority of students likely responded that they had encountered positive RMs in terms of contributions to the community because the formal curriculum provides students with the opportunity to interact with devoted community health care workers with excellent clinical competency and personalities who demonstrate such qualities. In this case, the curriculum likely increased the chance of encountering positive RMs and the likelihood of positive student perceptions.

In this study, older age and holding a bachelor’s degree before admission to medical school appear to have been influential factors in encountering fewer positive and more negative RMs. These students may have established their values prior to starting medical education. Encounters with negative RMs led to maintenance of their own values, as indicated by White in regard to “self-authorship” [21]. This suggests the possibility that older students and bachelor holders may face the challenge of strong value conflict as well as dissatisfaction with instructors and their learning environment. Those students must repeatedly negotiate between their pre-existing personal values and socially and professionally required desired values.

Although both male and female students had equivalent opportunities to encounter positive and negative RMs in formal clinical training courses, some gender differences, such as fewer negative RM encounters reported by female students, were identified. About 88 % of the faculty members of the Kagoshima University Faculty of Medicine were male in 2014, and the majority of instructors on clinical rotation were also male. This poses the question of whether female students applied their own personal values to male instructors or recognized male instructors as personal RMs. Furthermore, whether female students tend to accept professional values and compromise their own values more easily than their male counterparts remains unclear. It is also unclear whether RMs behaved better in front of female students. Stratton et al. reported that both male and female third-year medical students in a southeastern US medical school assigned roughly the same overall ranking to unprofessional behaviors of medical students, residents, and attending physicians [24]. Gude et al. reported that, compared with their male counterparts, female students had trouble identifying themselves as doctors, and therefore proposed that female students need to have female RMs [25]. The presence of gender differences in role modeling and professional development, perceptions of negative behavior or attributes, and value modification through interactions with RMs, need to be investigated in future studies, in addition to the lack of female RMs and the attributes of female students in medical education [26].

Medical educators need to consider the diversity among medical students, such as prior personal experiences and their resulting developmental statuses and values, and to facilitate conflict resolution compatible for individual levels of personal and professional development, even if they have same ethnic background and learn the same formal curriculum.

This study did have several limitations. First, the study sample only included one university in Japan, and the response rate was low; therefore, some care should be taken when generalizing the results. Second, data were based on student memories, and only one item was used for recognition of self-achievement, and therefore may not be reliable. Third, the RM categories and clinical situations used in this study were preset, and therefore may not cover the full range of RMs and situations. Further research is needed to confirm whether the differences and influential factors revealed in this research are applicable to other ethnicity groups, curriculums, and stages of medical education.

## Conclusions

Even if students have the same ethnic background and are learning the same curriculum, male students had more negative perceptions about their RMs compared with their female counterparts. In addition, older and more experienced students reported encountering more negative RMs, especially in terms of teaching-related behaviors.

Although most medical students encounter both positive and negative RMs during undergraduate medical education, the findings from this study suggest that in a variety of aspects, especially in terms of RM behaviors and attributes related to patient relationships, lifestyle, and teaching, encountering not only positive RMs, but also negative RMs, might facilitate student learning.

## Abbreviation

RM, role model

## Additional file

Additional file 1: Self-administered questionnaire regarding medical students’ role model encounters. (DOC 49 kb)
