# Supplementary material for: Effects of undergraduate medical students’ individual attributes on perceptions of encounters with positive and negative role models
Source: BMC Med Educ. 2016 Jun 23;16:164. doi: 10.1186/s12909-016-0686-1 (PMC4918193; doi:10.1186/s12909-016-0686-1)
Supplement: Additional file 1: — Self-administered questionnaire regarding medical students’ role model encounters. (DOC 49 kb) [file 12909_2016_686_MOESM1_ESM.doc]

Additional file 1: Self-administered questionnaire regarding medical students’ role model encounters

Please check the boxes that correspond to your feelings about whether you observed good or bad role model behavior from medical doctors in the following situations:

| Category of behaviors & attributes | Context of encounter |  | |
| --- | --- | --- | --- |
|  |  | “Good role model behavior” | “I will never behave like that” |
| a. Relationship with patients | Formal clinical training courses | □ | □ |
|  | Others | □ | □ |
|  |  |  |  |
| b. Clinical expertise | Formal clinical training courses | □ | □ |
|  | Others | □ | □ |
|  |  |  |  |
| c. Humanity, personal attributes | Formal clinical training courses | □ | □ |
|  | Others | □ | □ |
|  |  |  |  |
| d. Lifestyle | Formal clinical training courses | □ | □ |
|  | Others | □ | □ |
|  |  |  |  |
| e. Teaching students and health care professionals | Formal clinical training courses | □ | □ |
|  | Others | □ | □ |
|  |  |  |  |
| f. Contributions to the community | Formal clinical training courses | □ | □ |
|  | Others | □ | □ |
